# Discrimination between pathogenic and non-pathogenic *E. coli* strains by means of Raman microspectroscopy

**DOI:** 10.1007/s00216-020-02957-2

**Published:** 2020-10-08

**Authors:** Björn Lorenz, Nairveen Ali, Thomas Bocklitz, Petra Rösch, Jürgen Popp

**Affiliations:** 1 Institute of Physical Chemistry and Abbe Center of Photonics, Friedrich Schiller University Jena, Helmholtzweg 4, 07743 Jena, Germany; 2 InfectoGnostics Research Campus Jena, Philosophenweg 7, 07743 Jena, Germany; 3 Leibniz Institute of Photonic Technology Jena Member of the Research Alliance “Leibniz Health Technologies”, Albert-Einstein-Straße 9, 07745 Jena, Germany

**Keywords:** *E. coli*, Pathogen, Identification, Raman microspectroscopy, Bacterium, Single cell

## Abstract

Bacteria can be harmless commensals, beneficial probiotics, or harmful pathogens. Therefore, mankind is challenged to detect and identify bacteria in order to prevent or treat bacterial infections. Examples are identification of species for treatment of infection in clinics and *E. coli* cell counting for water quality monitoring. Finally, in some instances, the pathogenicity of a species is of interest. The main strategies to investigate pathogenicity are detection of target genes which encode virulence factors. Another strategy could be based on phenotypic identification. Raman spectroscopy is a promising phenotypic method, which offers high sensitivities and specificities for the identification of bacteria species. In this study, we evaluated whether Raman microspectroscopy could be used to determine the pathogenicity of *E. coli* strains. We used Raman spectra of seven non-pathogenic and seven pathogenic *E. coli* strains to train a PCA-SVM model. Then, the obtained model was tested by identifying the pathogenicity of three additional *E. coli* strains. The pathogenicity of these three strains could be correctly identified with a mean sensitivity of 77%, which is suitable for a fast screening of pathogenicity of single bacterial cells.

**Electronic supplementary material:**

The online version of this article (10.1007/s00216-020-02957-2) contains supplementary material, which is available to authorized users.

**Figure:**
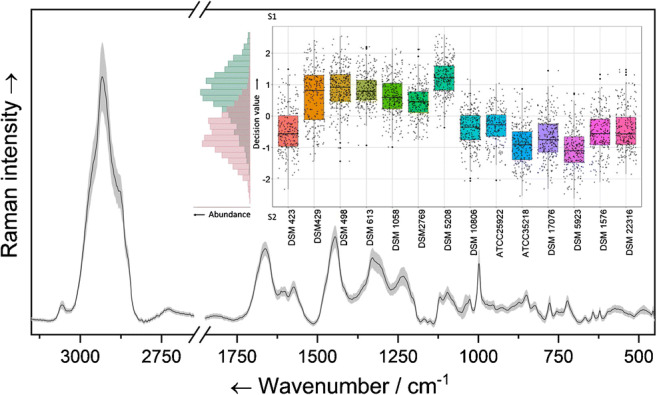
Graphical abstract

## Introduction

Bacteria are omnipresent in soil, water, food, and human and animal intestines and on the skin. Although the influence of bacteria on humans is mostly harmless, some bacteria can cause diseases. Two important routes of infection are through food and water. In 2010, foodborne pathogens including viruses caused worldwide 582 million events of foodborne illnesses and resulted in 351 thousand cases of death [1]. Consequently, identification of pathogenic bacteria is crucial in the prevention and treatment of infections. Nowadays, culture-based approaches still play a major role in identification of bacteria despite the drawback of long turnaround times, which may reach up to days [2–4]. Some bacteria species are predominantly pathogenic or non-pathogenic. In the case of blood cultures, the bacterial species can be used to estimate whether the isolated bacteria species is a pathogen causing the infection or a sample contamination, which, for example, originate from the skin [3]. For instance, *Pseudomonas aeruginosa*, *Streptococcus pneumoniae*, and *Klebsiella pneumoniae* are typically pathogenic [3].

It is not always reasonable to check for every possible pathogen. Instead, a suitable approach is sometimes to check for the presence of a limited number of pathogens which are the most frequent agents. Two methods to detect a selection of possible pathogens are PCR and microarrays. The benefit of microarrays and PCR is a low detection threshold, which enables the omission of long cultivation [5].

Sometimes only one bacterial species is of interest, especially if the species is used as an indicator. In the case of water, one source of contamination is feces. Here, the detection of *E. coli* serves as an overall indicator of water quality for fecal contamination [6].

However, *E. coli* can sustain in the environment for months, whereby naturalized *E. coli* are possibly harboring virulence genes and antibiotic resistances [6]. Further, *E. coli* itself is not necessarily a pathogen. *E. coli* Nissle 1917 for example is harmless and used for bowel remediation [7]. The example of *E. coli* shows that the identification of bacterial species is limited for risk assessment and an additional screening for pathogenicity may be required. Besides *E. coli*, *Mycobacterium* ssp*.* and *Legionella* ssp*.* are genera with closely related pathogenic and non-pathogenic species [8, 9], which requires discrimination of pathogenic and non-pathogenic strains.

Methods to detect specific pathogenic bacteria often examine the presence of virulence genes. Virulence factor genes can sometimes also be present in commensal bacteria, whereby the virulence factor genes are suppressed by other genes, and the bacterium is non-pathogenic [10]. Loss of the suppressing genes can turn a commensal into a pathogen [10]. Consequently, detection of virulence genes may not always be sufficient to determine pathogenicity. Environmental, food, and water samples are likely to contain new bacterial subspecies with unknown pathogenicity status. This can comprise a challenge for pathogen identification as some methods rely on targeting only specific virulence factors and other causes of virulence could be missed [2, 10].

While some identification methods reduce the task to detect specific genes, other technologies provide different features of whole bacterial cells. One of these technologies that offer a collection of biomolecular information within bacterial cells is Raman microspectroscopy. The spectroscopic fingerprint of the bacterial cell reflects the phenotype of the cell without qualitative and quantitative measuring of single chemical components. The obtained bacterial spectra are excellent data for bacterial identification [11]. For example, Hamasha et al. compared two chemometric methods to discriminate three non-pathogenic *E. coli* and one pathogenic *E. coli* [12]. However, many pathogenic and non-pathogenic strains are required to prove the possible discrimination between pathogenic and non-pathogenic strains as with only one strain for one group the identification problem simplifies to a strain identification.

Raman microspectroscopy has proven to yield high accuracies at the identification of pathogenic and non-pathogenic species as shown by Kusic et al. for *Legionella* ssp. and by Stöckel et al. for *Mycobacteria* spp. [8, 9].

Here, we apply Raman microspectroscopy to discriminate between pathogenic and non-pathogenic *E. coli* strains. First, we build a support vector machine model based on seven non-pathogenic *E. coli* and seven pathogenic *E. coli* strains. Second, we use our classifier to identify the pathogenicity of three additional *E. coli* strains.

## Materials and methods

Fourteen *E. coli* strains from strain collections (German Collection of Microorganisms and Cell Cultures GmbH, American Type Culture Collection) and three other *E. coli* strains have been cultured prior to classification and identification, respectively. Classification covers *E. coli* DSM 423, *E. coli* DSM 429, *E. coli* DSM 498, *E. coli* DSM 613, *E. coli* DSM 1058, *E. coli* DSM 1576, *E. coli* DSM 2769, *E. coli* DSM 5208, *E. coli* DSM 5923, *E. coli* DSM 10806, *E. coli* DSM 17076, *E. coli* DSM 22316, *E. coli* ATCC 25922, and *E. coli* ATCC 35218. Identification strains (referred to as isolates) comprise two pathogenic *E. coli* species isolated at the University Hospital Jena (here referred to as UK001 and UK002) and the non-pathogenic *E. coli* Nissle 1917, which is used for bowel remediation, e.g., Mutaflor®.

All *E. coli* species were incubated over night at 37 °C on Tryptic Soy Yeast Agar (TSY) which consists of 12 g Tryptic Soy Broth (Merck), 1.2 g Yeast extract (Sigma), and 6.0 g Agar (Sigma) per 400 ml deionized water and was adjusted to pH 7.

Sample preparation started with placing one inoculation loop of bacteria in 1 ml deionized water. Subsequently, the bacterial solution was washed three times. Each washing step consisted of a centrifugation step (10,000*g*, 1 min, 4 °C), discarding of the supernatant, and adding 1 ml deionized water. Washed bacteria suspension was deposited onto nickel foil (Rap. ID, Berlin; 0.25 mm thickness) and air-dried, yielding single measurable bacteria cells.

Bacteria cells were measured with a Bio Particle Explorer (rap. ID Particle System GmbH, Berlin Germany). Briefly, a solid-state frequency doubled Nd:YAG laser (LCM-s-111-NNP25, Laser export Co, Ltd) with a laser wavelength of 532 nm serves as the laser source. After the × 100 objective (MPLFLN-BD, Olymbus), the laser power is approximately 10 mW. The Raman signal is dispersed with a single-stage monochromator (HE532, Horiba Jobin Yvon, grating 920 lines/mm) and captured with a thermoelectrically cooled CCD (DV 401_BV). The final spectrum has a spectral resolution of 8 cm^−1^.

Single *E. coli* cells were measured with 50% laser power for 20 s. Within the 20 s, two spectra with 10 s were acquired from each cell. Overall, 3413 spectra pairs were acquired for the classification and 449 spectra pairs for the identification. Spectra of the classification strains are distributed on three independent biological replicates, while spectra of the isolates are distributed on two independent biological and technical replicates.

The obtained Raman spectra were pre-processed and analyzed based on in-house written functions using the statistical programing language R version 3.4.2 [13]. Our pre-processing pipeline started by despiking the spectra using a median filter followed by spectral wavenumber calibration [14] where all spectra were aligned between 300 and 3100 cm^−1^. The last two steps of spectral pre-processing were the baseline correction [15] and area normalization. Therein, the effect of background was extracted based on the iterative restricted least-squares (IRLS) algorithm; then, the corrected spectra were normalized.

After spectral pre-processing, a principal component analysis (PCA) model was combined with a support vector machine (SVM) model in order to reduce the high dimensionality of Raman spectra and to differentiate between the pathogenic and non-pathogenic bacteria. In this context, different chemometric techniques can be also implemented for feature selection and pathogenicity identification, e.g., biomolecular component analysis [16] and the combination of fuzzy principal component analysis or principal component analysis with linear discriminant analysis [17, 18]. In our work, the PCA-SVM model was trained on the 14 cultivated *E. coli* strains while the pathogenicity of the *E. coli* isolates was predicted using the trained model. Within the training part, the classification mean sensitivity was calculated based on different numbers of principal components (PCs) and using a radial kernel that was optimized for different values of the cost parameter and the tolerance parameter (Figs. S1 and S2, see Electronic Supplementary Material, ESM). In ESM Fig. S2, the results of parameter optimization based on the leave-one-strain-out cross-validation and for a classification tolerance of the value 1 are presented. Leave-one-strain-out cross-validation [19] was chosen to take into account the strain to strain variability. The classification is always done on a subset which is a different strain than the rest on the model. This prevents classification of a strain as pathogenic or non-pathogenic only because the strain is contained in the subset and the rest at the same time. Based on our optimization search, the best classification performance is achieved when the SVM model is constructed on 22 PCs for a 0.5 classification cost and 1 classification tolerance. The classification mean sensitivity in this case is around 81.2%. Parameter optimization takes around 470 min, but then can be used to identify new data sets without new parameter optimization. After parameter optimization, the constructed model was implemented to differentiate between the pathogenic and non-pathogenic strains of the isolates data set, which took around 3 min.

Alternative statistics based on a leave-one-batch-out cross-validation is presented in addition in the ESM. The additional ESM contains parameter optimization (Fig. S3), spectra distribution along decision value for model validation and identification (Figs. S4 and S5), and validation and identification sensitivities (Tables S3 and S4).

## Results and discussion

**Fig. 1:**
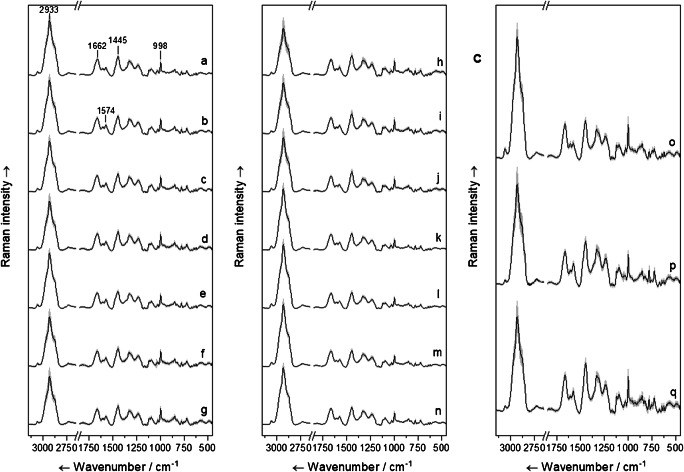
Mean Raman spectra of non-pathogenic *E. coli* on the left (a–g) and pathogenic *E. coli* in the middle (h–n) and the “isolates” on the right (o–q). Non-pathogenic *E. coli* strains DSM 423 (a), DSM 429 (b), DSM 498 (c), DSM 613 (d), DSM 1058 (e), DSM 2769 (f), and DSM 5208 (g). Pathogenic *E. coli* strains DSM 10806 (h), ATCC 25922 (i), ATCC 35218 (j), DSM 17076 (k), DSM 5923 (l), DSM 1576 (m), and DSM 22316 (n). Finally, mean spectra of *E. coli* Nissle 1917 (o), UK013 (p), and UK014 (q)

We use for our classification seven non-pathogenic and seven pathogenic *E. coli* strains. The Raman mean spectra of the strains which are used for classification are depicted in Fig. 1a–g and h–n. Mean spectra show the common and expected bands for *E. coli*, which are mainly CH stretching vibrations at 2933 cm^−1^, the amide I band at 1662 cm^−1^, the adenine/guanine band at 1574 cm^−1^, the CH_2_ and CH_3_ deformation vibrations at 1445 cm^−1^, and the phenylalanine ring breathing vibration at 998 cm^−1^. A comparison of Raman mean spectra of the non-pathogenic *E. coli* strains (Fig. 1a–g) with the pathogenic *E. coli* strains (Fig. 1h–n) does not show significant differences for the observer. The similarity of the *E. coli* mean spectra has been expected because all strains belong to the same species. The same is true for the isolates (Fig. 1o–q) used as independent strains to be identified. Our isolates include two *E. coli* strains which originate from patients and *E. coli* Nissle 1917, which is widely used for bowel remediation. The clinical isolates are pathogenic strains, while Nissle 1917 is a non-pathogenic strain.

Because of the close biological relationship and the resulting high similarity between the Raman spectra, we implement a support vector machine model to assess whether pathogenicity can be identified at strain level by Raman microspectroscopy. Further, our scientific question is a two-class problem. Two-class problems are prone to show possible distinguishability by multivariate statistics no matter the presence or non-presence of the discriminability. We use two important measures to ensure a reliable answer, whether we can distinguish between pathogenic and non-pathogenic strains. First, we use a large group of pathogenic and non-pathogenic strains. Thereby, our classifier comprises strain to strain variability. This ensures that we do not just measure the strain to strain variability of two strains and assume this difference is derived from pathogenicity, but we actually test for a difference caused by pathogenicity. Second, we use independent strains as validation of our classifier, which are not utilized for constructing the classifier; thus, the classifier was not trained using the pathogenicity of the test strains.

**Fig. 2:**
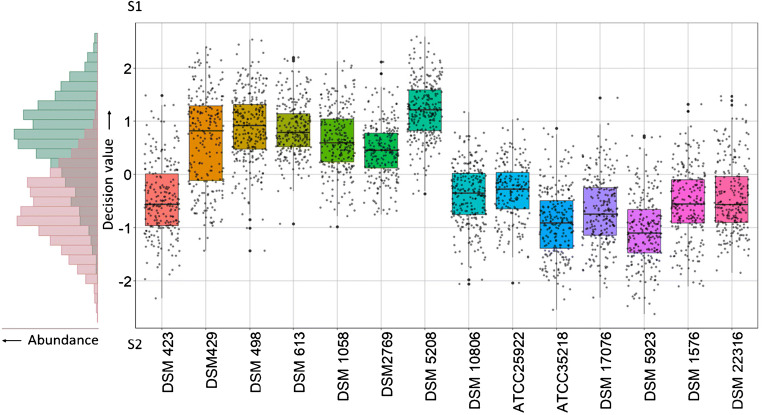
Distance from the classification border of the SVM. Classification shown as histogram, single spectra, strain spectra median, strain spectra standard deviation, and first and third quantiles (boxes). Classification involving non-pathogenic *E. coli* strains DSM 423, DSM 429, DSM 498, DSM 613, DSM 1058, DSM 2769, and DSM 5208, and pathogenic *E. coli* strains DSM 10806, ATCC 25922, ATCC 35218, DSM 17076, DSM 5923, DSM 1576, and DSM 22316

The SVM classification as well as the pathogenicity assignment of the isolates is visualized in Fig. 2 (and in ESM Fig. S4 with an alternative cross-validation). The left side of Fig. 2 shows the histogram of SVM decision values of the non-pathogens’ and pathogens’ spectra, respectively. The non-pathogens’ spectra are mainly distributed at positive decision values with a maximum around + 0.8 while the pathogens’ spectra are mainly distributed at negative decision values with a maximum around − 0.7. Both histograms overlap but can be separated. Overall, the curves have a wide spread, which implies a large variance between the spectra. This variance is expected since the measured single bacteria cells are not cloned. The histogram distribution of the non-pathogens’ spectra has a tail towards negative decision values indicating some sort of unexpected misclassification. This unexpected misclassification will be visible at the single strains’ distribution, as shown in Fig. 2 on the right.

The right side of Fig. 2 displays the spectra of every strain of the classifier against the corresponding decision value of the SVM. The first seven strains from the left are non-pathogenic *E. coli* while the remaining seven strains on the right correspond to pathogenic *E. coli*. A positive decision value means classification as non-pathogenic, whereas a negative value corresponds to pathogenic bacteria. Most non-pathogenic strains’ bacteria are correctly assigned as non-pathogenic (six out of seven), while eight strains’ including one non-pathogenic strain’s bacteria are classified as pathogenic. *E. coli* DSM 423 is the non-pathogenic strain which is predominantly assigned as pathogenic and is responsible for the earlier observed tail in the histogram. Overall, 81.1% of the spectra are correctly classified according to their pathogenicity. The non-pathogenic strains yielded a sensitivity of 80.1%; likewise, a sensitivity of 82.2% was obtained for the pathogenic strains.

**Fig. 3:**
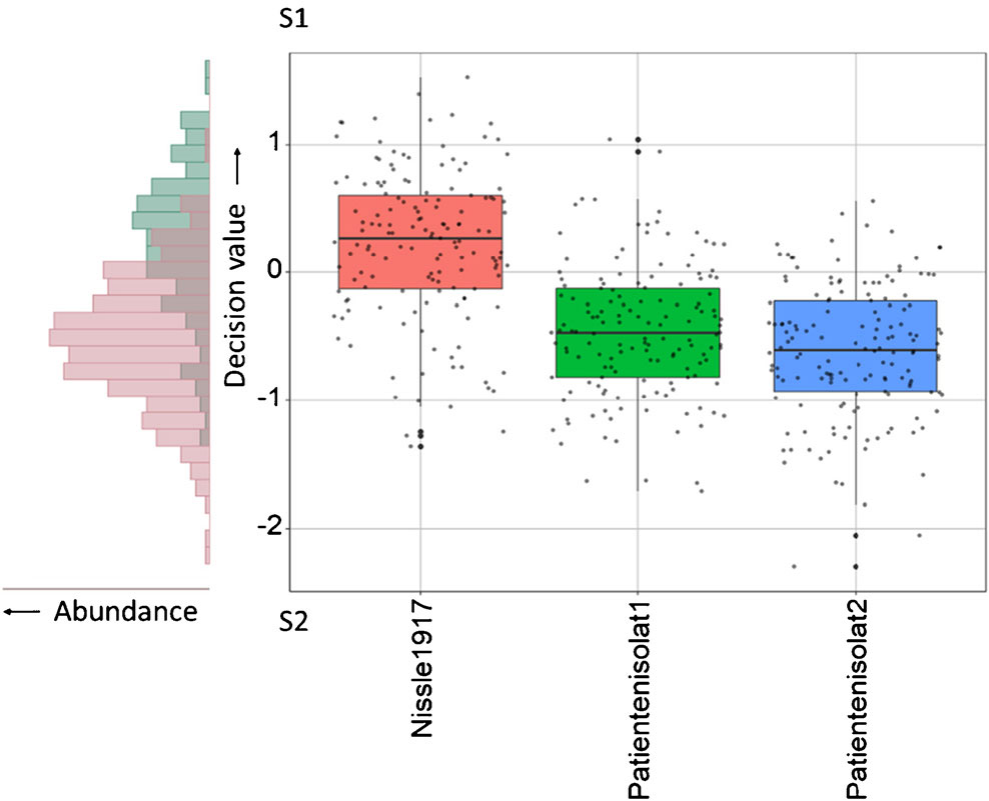
Distance from the classification border of the SVM. Identification shown as histogram, single spectra, strain spectra median, strain spectra standard deviation, and first and third quantiles (boxes). Identification enclosing the patient isolates 1 and 2 and *E. coli* Nissle 1917

The classification indicates an ability to distinguish pathogenicity of *E. coli* strains by Raman microspectroscopy. We use the previously mentioned isolates to validate the identification of *E. coli*’s pathogenicity (Fig. 3). The spectra are again allocated in respect to the decision values of the trained SVM as histogram and the isolate strains. Therefore, positive values are identified as non-pathogen and negative values as pathogen. Within the histogram, *E. coli* Nissle 1917 is mainly at positive decision values, whereby the spectra are assigned to decision values between − 1.4 and 1.7. The mode of the decision value is at 0.4. This is about 0.3 lower mode than the non-pathogen spectra of the classifier. One possible explanation is that the preferred habitat of *E. coli* Nissle 1917 is the bowel. Contrary, the non-pathogen strains of the classifier have been maintained over long time within strain collections. One limitation of Raman microspectroscopy is often not to be able to trace back unexpected interferences or the biological reason for the result on real-world samples, like the pathogenicity trait of a new strain. This disadvantage is counterbalanced by the missing prerequisite for genetic information, e.g., target genes. The pathogen spectra have their mode at the decision value − 0.5 with a spread between − 2.3 and 1.1. However, pathogens’ distribution in the histogram is similar to the classification, thus supporting a reliable validation. The distributions are not separated in the histograms, because the distribution of the non-pathogenic *E. coli* has a comparable smaller sample size compared with the pathogenic. A separation can be seen in the plot of the decision values of the identification against the strains (Fig. 3, right side). It shows the expected variance of the pathogen spectra. Overall, both clinical isolates are identified with a sensitivity of 86.3% as pathogenic, while the non-pathogenic *E. coli* Nissle 1917 is identified with a sensitivity of 67.1% as non-pathogenic. Similar results to Fig. 3 are shown by ESM Fig. S5, which is based on an alternative model cross-validation.

In some cases, it can be assumed that the sample contains only one species. If so, the combination of several spectra can increase the obtained sensitivities, as the majority vote counterbalance misclassification originating through the large variance between spectra. While the regular classification yielded a mean sensitivity of 81.2%, the majority vote combining 3, 5, or 7 spectra increases the mean sensitivity to 87.7, 90.0, and 92.0% for the cultivated Raman spectral data set (Table S1, see ESM). Similarly, the mean sensitivity for the pathogenicity identification of isolate data set increases from 76.7% to 86.9, 91.3, and 94.8% for the majority vote combining 3, 5, or 7 spectra (Table S2, ESM). Results of the majority vote with an alternative model cross-validation are shown in Tables S3 and S4 in the ESM.

## Conclusion

We evaluated Raman microspectroscopy for its potential to discriminate pathogenic from non-pathogenic *E. coli* strains. 86.3% cells of two clinical pathogenic isolates are correctly identified as pathogenic while 67.1% cells of the non-pathogenic *E. coli* Nissle 1917 are correctly identified as non-pathogenic. Notably, *E. coli* Nissle 1917 is reported to have several characteristics which are relevant for its probiotic nature [7]. These reported characteristics discern *E. coli* Nissle 1917 from both pathogenic and non-pathogenic *E. coli* [7].

Notably, our approach does not include these three strains in our reference data set, which comprises seven pathogenic and seven non-pathogenic *E. coli* strains each. Our aim is to identify *E. coli* pathogenicity, and we have to be cautious not to misinterpret strain identification for pathogenicity identification. One safeguard is the use of the three additional strains for identification. The other safeguard is the increased strain variety by using 14 strains for the classifier. The result of the identification alongside the two provisions for reliable results shows that Raman microspectroscopy is a suitable tool to predict pathogenicity of *E. coli* strains. Even better prediction can be achieved, if a majority vote of multiple spectra is used (Table S1 and S2, see ESM). In this manner, the identification reaches mean sensitivities of 86.9, 91.3, and 94.8%, if 3, 5, and 7 spectra are used to generate a prediction, respectively.

Although Raman microspectroscopy enables the prediction of pathogenicity in our study, we do not expect it to be able to specify pathogenicity traits, like virulence genes. Raman microspectroscopy is a phenotypic method. As originating from phenotypic method, Raman spectra are a superposition of spectral information of all biomolecules within the bacterial cell. Bacterial Raman spectra do not verify which biological pathways and biomolecules are presented and responsible for the pathogenicity. Thus, investigation of pathogenicity by Raman microspectroscopy has to be followed up by other techniques, if the mechanism of the virulence should be specified.

Raman microspectroscopy could fit in the niche of fast pathogenicity estimation without the expenses of sequencing or prior knowledge of expected virulence genes. The advantage of Raman spectroscopy could be pathogenic screening without prior genetic understanding of the bacteria to be investigated either in combination with selective agar or species identification by Raman spectroscopy to pinpoint the species before. For further studies, we recommend using isolates instead of strain collections’ strains for the classifier. This could further improve the potential accuracy of pathogenicity screening in the intended field of application.

## Electronic supplementary material

ESM 1 (PDF 699 kb)

## References

[1] Kirk MD, Pires SM, Black RE, Caipo M, Crump JA, Devleesschauwer B (2015). World Health Organization estimates of the global and regional disease burden of 22 foodborne bacterial, protozoal, and viral diseases, 2010: a data synthesis. PLoS Med.

[2] Zhao X, Lin CW, Wang J, Oh DH (2014). Advances in rapid detection methods for foodborne pathogens. J Microbiol Biotechnol.

[3] Weinstein MP, Towns ML, Quartey SM, Mirrett S, Reimer LG, Parmigiani G (1997). The clinical significance of positive blood cultures in the 1990s: a prospective comprehensive evaluation of the microbiology, epidemiology, and outcome of bacteremia and fungemia in adults. Clin Infect Dis.

[4] Valérie T. Review of methods for the rapid identification of pathogens in water samples - ERNCIP thematic area Chemical & Biological Risks in the Water Sector - Task 7, deliverable 1. Rep EUR. 2014;26881. 10.2788/18775.

[5] Ramirez-Castillo FY, Loera-Muro A, Jacques M, Garneau P, Avelar-Gonzalez FJ, Harel J (2015). Waterborne pathogens: detection methods and challenges. Pathogens..

[6] Jang J, Hur HG, Sadowsky MJ, Byappanahalli MN, Yan T, Ishii S (2017). Environmental Escherichia coli: ecology and public health implications-a review. J Appl Microbiol.

[7] Sonnenborn U, Schulze J (2009). The non-pathogenic Escherichia coli strain Nissle 1917: features of a versatile probiotic. Microb Ecol Health Dis.

[8] Stöckel S, Meisel S, Lorenz B, Kloß S, Henk S, Dees S (2017). Raman spectroscopic identification of Mycobacterium tuberculosis. J Biophotonics.

[9] Kusic D, Kampe B, Rösch P, Popp J (2014). Identification of water pathogens by Raman microspectroscopy. Water Res.

[10] Merhej V, Georgiades K, Raoult D (2013). Postgenomic analysis of bacterial pathogens repertoire reveals genome reduction rather than virulence factors. Brief Funct Genomics.

[11] Lorenz B, Wichmann C, Stöckel S, Rösch P, Popp J (2017). Cultivation-free Raman spectroscopic investigations of bacteria. Trends Microbiol.

[12] Hamasha K, Mohaidat QI, Putnam RA, Woodman RC, Palchaudhuri S, Rehse SJ (2013). Sensitive and specific discrimination of pathogenic and nonpathogenic Escherichia coli using Raman spectroscopy—a comparison of two multivariate analysis techniques. Biomed Opt Express.

[13] R Development Core Team (2017). R: a language and environment for statistical computing.

[14] Dörfer T, Bocklitz T, Tarcea N, Schmitt M, Popp J (2011). Checking and improving calibration of Raman spectra using chemometric approaches. Z Phys Chem.

[15] Guo S, Bocklitz T, Popp J (2016). Optimization of Raman-spectrum baseline correction in biological application. Analyst..

[16] Kuzmin AN, Pliss A, Prasad PN. Ramanomics: new omics disciplines using micro Raman spectrometry with biomolecular component analysis for molecular profiling of biological structures. Biosensors (Basel). 2017;7(4). 10.3390/bios7040052. 10.3390/bios7040052 PMC5746775 29140259

[17] Dina NE, Gherman AMR, Chis V, Sarbu C, Wieser A, Bauer D (2018). Characterization of clinically relevant fungi via SERS fingerprinting assisted by novel chemometric models. Anal Chem.

[18] Gherman AMR, Dina NE, Chis V, Wieser A, Haisch C (2019). Yeast cell wall - Silver nanoparticles interaction: a synergistic approach between surface-enhanced Raman scattering and computational spectroscopy tools. Spectrochim Acta A Mol Biomol Spectrosc.

[19] Guo S, Bocklitz T, Neugebauer U, Popp J (2017). Common mistakes in cross-validating classification models. Anal Methods.

