# Supplementary material for: Discrimination between pathogenic and non-pathogenic E. coli strains by means of Raman microspectroscopy
Source: Anal Bioanal Chem. 2020 Oct 8;412(30):8241–7. doi: 10.1007/s00216-020-02957-2 (PMC7680742; doi:10.1007/s00216-020-02957-2)
Supplement: Supplementary file 1 — (PDF 699 kb) [file 216_2020_2957_MOESM1_ESM.pdf]

Analytical and Bioanalytical Chemistry

Electronic Supplementary Material

Discrimination between pathogenic and non-pathogenic *E. coli* strains by means of Raman microspectroscopy

Björn Lorenz, Nairveen Ali, Thomas Bocklitz, Petra Rösch, Jürgen Popp

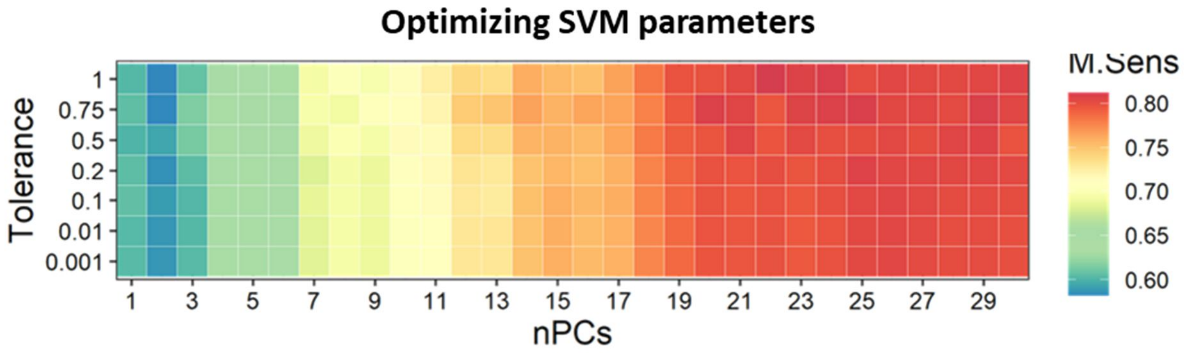

Fig. S1 Optimization of SVM parameter, tolerance. The mean sensitivity of SVM is shown as heat map depending on the tolerance parameter of the SVM model and the number of PCs

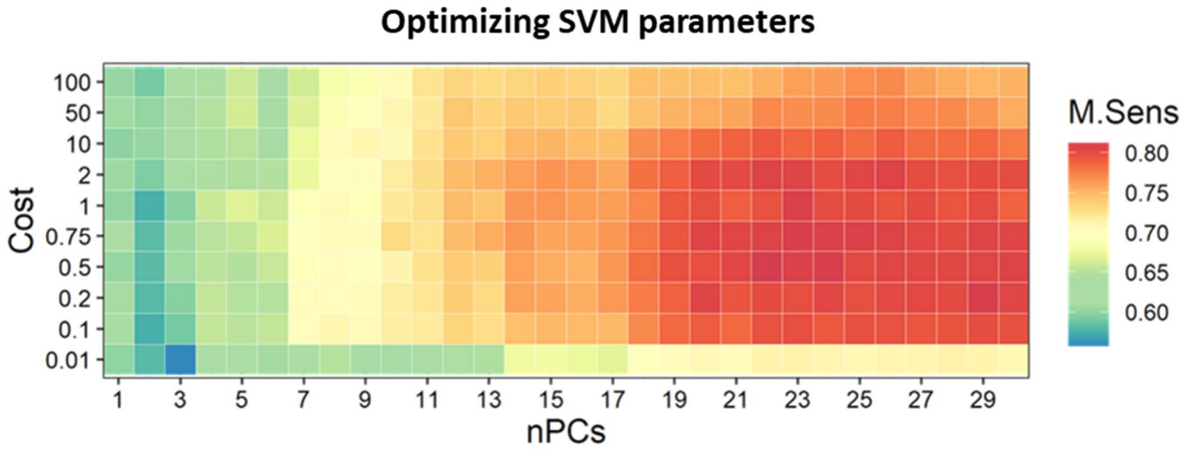

Fig. S2 Optimization of SVM parameter, cost. The mean sensitivity of SVM is shown as heat map depending on the cost parameter of the SVM model and the number of PCs

Table S1 Sensitivities for (non-)pathogenic bacteria and overall mean sensitivities of the classification with no majority vote (number of bacteria 1) and majority vote (number of bacteria; 3, 5, 7)

CLASSIFICATION

NUMBER OF BACTERIA	Sensitivity in %		Mean Sensitivity in %
	Non-pathogenic	Pathogenic	
**1**	80.2	82.2	81.2
**3**	85.0	90.5	87.7
**5**	86.2	94.2	90.2
**7**	86.8	97.2	92.0

Table S2 Sensitivities for (non-)pathogenic bacteria and overall mean sensitivities of the identification with no majority vote (number of bacteria 1) and majority vote (number of bacteria; 3, 5, 7)

IDENTIFICATION

NUMBER OF BACTERIA	Sensitivity in %		Mean Sensitivity in %
	Non-pathogenic	Pathogenic	
**1**	67.1	86.3	76.7
**3**	79.2	94.7	86.9
**5**	83.9	98.7	91.3
**7**	89.9	99.7	94.8

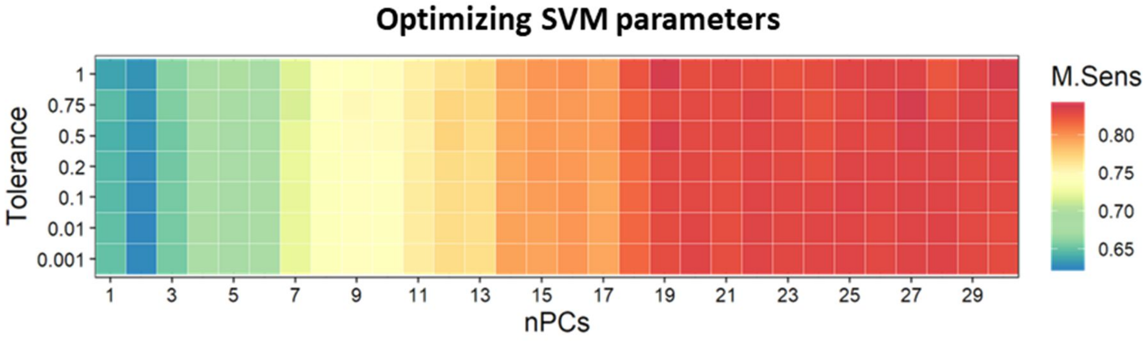

Fig. S3 Optimization of SVM parameter, tolerance. The mean sensitivity of SVM is shown as heat map depending on the tolerance parameter of the SVM model and the number of PCs. Here, classification is validated by a Leave-one-batch-out cross-validation instead a Leave-one-strain-out cross-validation

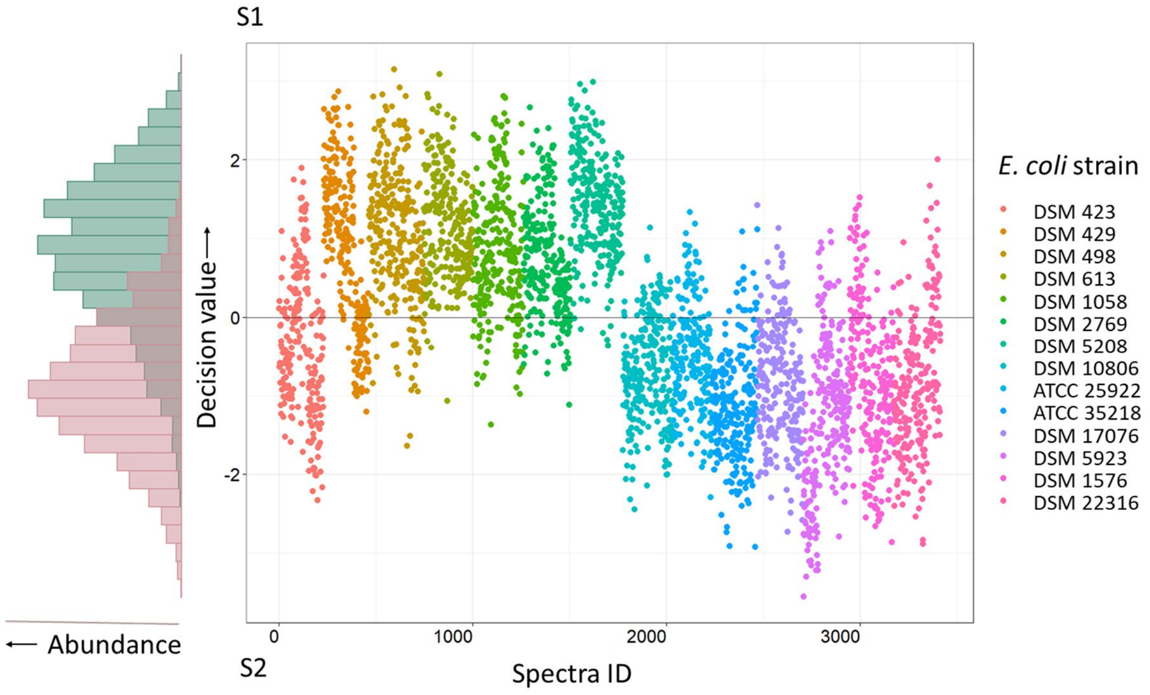

Fig. S4 Distribution of spectra along the decision value, overall, on the left side and for each spectra ID on the right side. Distribution was created by a PCA-SVM model using a Leave-one-batch-out cross-validation. PCA-SVM model was feed by nonpathogenic *E. coli* strains DSM 423, DSM 429, DSM 498, DSM 613, DSM 1058, DSM 2769, DSM 5208, and pathogenic *E. coli* strains DSM 10806, ATCC 25922, ATCC 35218, DSM 17076, DSM 5923, DSM 1576, DSM 22316

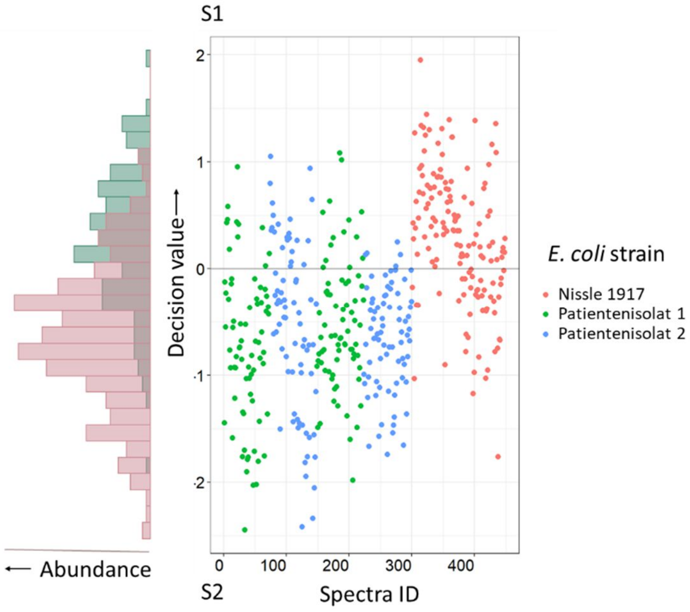

Fig. S5 Identification of two pathogenic *E. coli* from patients and *E. coli* Nissle 1917 by PCA-SVM model tested with a Leave-one-batch-out cross-validation. Depicted is the overall spectra distribution along the decision value on the left side and the decision value for each spectra ID on the right side

Table S3 Sensitivities for (non-) pathogenic bacteria and overall mean sensitivities of the classification with no majority vote (number of bacteria 1) and majority vote (number of bacteria; 3, 5, 7). Here, classification is validated by a Leave-one-batch-out cross-validation instead of a Leave-one-strain-out cross-validation

CLASSIFICATION

NUMBER OF BACTERIA	Sensitivity in %		Mean Sensitivity in %
	Non-pathogenic	Pathogenic	
**1**	81.9	85.4	83.6
**3**	86.4	94.3	90.4
**5**	88.3	97.6	92.9
**7**	88.3	98.72	93.5

Table S4 Sensitivities for (non-) pathogenic bacteria and overall mean sensitivities of the identification with no majority vote (number of bacteria 1) and majority vote (number of bacteria; 3, 5, 7). Here, PCA-SVM model was optimized by a Leave-one-batch-out cross-validation instead a Leave-one-strain-out cross-validation

IDENTIFICATION

NUMBER OF BACTERIA	Sensitivity in %		Mean Sensitivity in %
	Non-pathogenic	Pathogenic	
**1**	69.1	82.7	75.9
**3**	83.2	95.0	89.1
**5**	88.6	95.0	91.8
**7**	89.9	99.0	94.5
